# Investigation of the Relationship Between Sensory Processing Skills and Language Development in Children With Developmental Language Disorders

**DOI:** 10.1002/brb3.70105

**Published:** 2024-12-26

**Authors:** Elife Barmak, Banu Baş

**Affiliations:** ^1^ Department of Speech and Language Therapy, Faculty of Health Sciences Ankara Yıldırım Beyazıt University Ankara Turkey; ^2^ Department of Audiology, Faculty of Health Sciences Ankara Yıldırım Beyazıt University Ankara Turkey

**Keywords:** development language disorder, preschool period, sensory processing skills

## Abstract

**Background:**

The aim was to compare the sensory processing skills of children diagnosed with developmental language disorder (DLD) with those of typically developing children and to investigate the relationship between language development and sensory processing skills.

**Methods:**

The investigation comprised 60 children, all of whom were typically developing and diagnosed with DLD. The “Early Language Development‐Third: Turkish (ELD: Turkish)” and the “Sensory Profile (Caregiver Questionnaire)” were utilized to assess the language and sensory processing skills of the children, respectively, within the scope of the study.

**Results:**

In our study, the four‐factor scores of children with DLD were considerably lower than those of their typically developing peers (*p* < 0.05). These factors are sensory seeking, inattention/distractibility, fine motor/perceptual, and emotionally reactive. In addition, the children with DLD had statistically significant lower scores in three quadrants except for sensory sensitivity (*p* < 0.05). In three quadrants (sensation seeking, low registration, and sensory avoiding) and three factors (sensory seeking, inattention/distractibility, and fine motor/perceptual), a substantial correlation was observed between the sensory processing skills and the language skills.

**Conclusions:**

It has been observed that there are differences in the sensory processing skills of children with DLD. In addition to evaluating the language skills of these children, it will also contribute to the evaluation of the effectiveness of their sensory processing skills and the development of more effective strategies in the intervention processes of these children.

## Graphical abstract

Children with DLD had lower mean scores in three quadrants—low registration, sensory avoiding, and sensation seeking—than typically developing children. However, the mean sensory sensitivity scores of the two groups were not different.

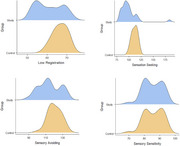

## 1 Introduction

Development language disorder (DLD) is a language disorder that arises during the developmental process and is not related to a recognized biomedical cause, such as intellectual disability, autism spectrum disorder (ASD), hearing loss, or genetic factors (Bishop et al. 2016, 2017). DLD is very common in early childhood (McGregor 2020; Norbury et al. 2016). Prevalence studies on DLD are reported to be around 1% to 7% (Calder et al. 2022; Norbury et al. 2016; Oyono, Pascoe, and Singh 2018; Tomblin et al. 1997).

Children with DLD may have problems with receptive and/or expressive language abilities. Expressive language is the capacity to accurately transmit thoughts and emotions into words and sentences in a grammatically and semantically correct manner. In contrast, receptive language is the capacity to comprehend the meanings of words and sentences (Plug et al. 2021). Phonology, morpho‐syntax, semantics, pragmatics, discourse, and verbal learning abilities affect the receptive and expressive language abilities of children with DLD (Bishop et al. 2017). Due to the effects on the basic components of the language, children experience difficulties in using, understanding, and learning the spoken language (McGregor 2020). It is suggested that the child may experience challenges in motor and other cognitive processes, as well as language development, due to the child's language development not progressing at the typical rate (Hill 2001). Therefore, DLD may be accompanied by disorders related to attention, motor, literacy, behavioral, and auditory processing disorders (Bishop et al. 2016, 2017). Furthermore, these situations give rise to emotional, behavioral, social, and academic challenges for these children (Christopulos and Redmond 2023).

The extent to which we can engage with our surroundings is contingent upon the capabilities of our cognitive system to recognize, utilize, and cohesively amalgamate diverse sensory stimuli (Ayres 1989). Ayres (1989) developed the sensory integration theory to explain the behavior of children with learning disabilities. This theory refers to a neurological process that organizes the senses coming from the individual's body and the environment and allows the body to use them effectively in the environment (Ayres 1989; Schaaf et al., 2010). Sensory processing describes the comprehensive mechanism through which the central and peripheral nerve systems regulate sensory input. This process involves the reception, modulation, integration, and structuring of inputs. Sensory integration is just one part of the entire sensory processing process (Miller and Lane 2000). Sensory processing disorder is a condition in which the brain has difficulty regulating and/or organizing behavioral responses to sensory input in line with environmental demands (participation in daily life routines and activities) (Miller et al. 2007, 2009). This condition may be correlated with learning, sensory, and developmental disabilities (Miller et al. 2007; Mitchell et al. 2015). Disorders in sensory processing have been reported in children with attention deficit hyperactivity disorder (ADHD) (Rani et al. 2023), ASD (Thye et al. 2018), and hearing impairment (Baş and Yücel 2023).

Bishop (2009) emphasizes that children with specific language disorders do not learn at a normal speed compared to their typically developing peers, so it is crucial to pay attention to learning mechanisms rather than focusing on perceptual or linguistic impairments as causal factors. Challenges with sensory processing can also be one of the processing problems that interfere with normal learning processes, especially language learning (Mandelbaum et al. 2006). It is stated in the literature that children without neurodevelopmental or mental disorders show differences in sensory processing (Mulligan, Douglas, and Armstrong 2021). Trauner et al. (2000) showed that 70% of children with DLD and 22% of healthy children had abnormal neurological functions. They emphasized that sensory deficits, fine motor impairments, hyperreflexia, and oral motor apraxia were among the neurological findings of children with language disorders. A study indicates that there is a relationship between language disorders and sensory processing skills (Casey 2016). However, it has also been stated that there is no significant relationship between the language skills and sensory processing skills of school‐age children with DLD. It has been determined that 60% of these children have differences in sensory processing (Simpson et al. 2020). Taal et al. (2013) emphasize that sensory processing systems are involved in language acquisition processes, and that impaired modulation of sensory input can also negatively affect language processing. Additionally, this study suggests that language therapy will greatly benefit children with language disorders, but more general intervention approaches will better support these children's general developmental functions.

It is critical to promote optimal development in all developmental domains for children with DLD throughout the preschool period, together with language therapy, to prevent any learning problems. We believe that identifying these children's disparities in sensory processing skills during their early years and providing co‐therapy for these skills along with language therapy will yield greater advantages for children in terms of learning, sensory, and developmental dimensions. In this context, our study aimed to reveal the differences by comparing the sensory processing skills of children diagnosed with DLD with those of their typically developing peers, as well as investigate the relationship between receptive and expressive language skills and sensory processing skills.

## 2 Materials and Methods

This research is a descriptive survey that aims to compare the sensory processing skills of children diagnosed with DLD to those of their peers. Additionally, it seeks to investigate the correlation between the language development and the sensory processing skills.

### 2.1 Population and Sample

The sample size was calculated using the G‐power program, and the study was completed with an 85% power (*α* = 0.05, effect size = 0.80). In this context, the sample of our research included 30 GDB children directed to special education and rehabilitation centers, regardless of gender, and 30 typically developing children in the same age group who were receiving education in independent preschools and volunteered to participate in the study. In our country, the child's difficulties/limitations in functional activity in language, speech, and communication and participation in life are evaluated by relevant specialists through family‐centered holistic clinical evaluations (developmental history taken from the family, observation of language, speech, and communication functions during free play activities, detailed physical examinations, and tests evaluating standard language–speech–communication skills). For children diagnosed with DLD as a result of the evaluation, if their receptive or expressive language development is between −1.5 and −3 SD or −3 SD and below, they are directed to receive education services in the field of speech, language, and communication for at least 1 year with the decision of specialists. In this context, the criteria for inclusion in our study are as follows: (a) being diagnosed with DLD; (b) the child should not have a language disorder due to an etiological cause such as a motor, neurological, hearing, or psychiatric disorder; and (c) being referred to a special education and rehabilitation center for language therapy or receiving language therapy.

For the typically developing group, children were required to show typical development. The study excluded children who did not satisfy these criteria.

### 2.2 Data Collection Tools

The research used the Early Language Development Test‐Third Edition: Turkish, and Sensory Profile Caregiver Survey as data‐gathering instruments.

#### 2.2.1 Early Language Development‐Third Edition: Turkish (ELD‐3: Turkish)

This test evaluates the receptive and expressive language skills of early children. It is a privately administered, norm‐referenced test whose validity and reliability are established by Güven and Topbaş (2014). This language test aims to evaluate the receptive and expressive language skills of children between the ages of 2 and 7 years and 11 months and to detect possible language disorders. The test consists of two subtests: “receptive language” and “expressive language.” There are a total of 76 items in each form. Some of these items involve showing pictures or describing, whereas others involve fulfilling verbal instructions and answering questions verbally (Güven and Topbaş 2014; Topbaş and Güven 2011). In the test, the child receives 1 point for each correct response and 0 point for each incorrect response. The raw score is obtained from the total score resulting from the test. The raw scores obtained are converted into standard scores according to the tables on the back of the test.

#### 2.2.2 Sensory Profile (Caregiver Questionnaire)

This profile is a 125‐item caregiver questionnaire to assess the sensory processing skills of children aged 3–10 years. The questionnaire was validated and reliable in Turkish by Kayıhan et al. (2015). It assesses the effects of sensory processing skills on the functional performance of a child's daily living activities, likewise the child's behavioral and emotional reactions (Dunn 1999; Kayıhan et al. 2015). This survey is divided into 14 sections and three main categories: (1) sensory processing (six sections), (2) modulation (five sections), and (3) behavioral and emotional responses (three sections). Nine factors are defined based on the principal component factor analysis results of the questionnaire's 125 items. These factors are classified based on children's reactions to sensory inputs in their sensory systems. These factors are sensory seeking (Factor 1), emotionally reactive (Factor 2), low endurance/tone (Factor 3), oral sensory sensitivity (Factor 4), inattention/distractibility (Factor 5), poor registration (Factor 6), sensory sensitivity (Factor 7), sedentary (Factor 8), and fine motor/perceptual (Factor 9). Four quadrants (low registration, sensation seeking, sensory sensitivity, and sensory avoiding) were created by combining the scores from specific factors and sections. Accordingly, while sensation seeking reflects high sensory thresholds with an active self‐regulation strategy, sensory avoiding reflects low sensory thresholds with an active self‐regulation strategy. Although sensory sensitivity is associated with low sensory thresholds with a passive self‐regulation strategy, low registration is associated with high sensory thresholds with a passive self‐regulation strategy (Dunn 1997; Ermer and Dunn 1998). Within the scope of the research, parents scored each item in the survey according to the frequency of their children's behavior (1 = always, 2 = often, 3 = sometimes, 4 = rarely, and 5 = never). The questionnaire includes low‐ and high‐threshold items. High‐threshold items measure the child's lack of response or need for more intense stimuli. Low‐threshold items measure the child's awareness, discomfort, and distress to sensory stimuli. Raw scores from the questionnaire were added to create the nine‐factor and the four‐quadrant scores. The average application time for the survey is 20–30 min. In our research, nine factors and four quadrants were examined.

### 2.3 Collection of Data

Before conducting the investigation, ethical approval was obtained from the Health Sciences Ethics Committee of Ankara Yıldırım Beyazıt University (date: December 9, 2021; protocol no. 33). The parents of the participants who consented to take part in the study provided informed consent after being apprised of the research objectives. After obtaining information about the child from the parents, the researcher asked the parents to fill out the questionnaire. In a room devoid of distractions and to assess the child's receptive and expressive language abilities, the researchers administered the language test.

### 2.4 Analysis of Data

All data in the research were initially recorded using the IBM SPSS 26.0 software suite. These data were subjected to a normality test to ascertain whether they adhered to a normal distribution. However, the final decision was used in the skewness and kurtosis coefficients. Accordingly, data with skewness and kurtosis coefficients between +1.5 and −1.5 (Tabachnick and Fidell 2013) are considered to be normally distributed, but data deviating from these values are considered to be non‐normally distributed. The study analyzed the data from a language test and sensory processing skills of children using independent *t*‐tests for those with normal distributions and Mann–Whitney *U* tests for those with non‐normal distributions. The distribution of the groups in terms of gender was analyzed with the chi‐square test. The relationship between the nine‐factor and the four‐quadrant scores of sensory processing skills and the receptive and expressive language skills was tested by correlation analysis.

## 3 Results

The study consisted of 30 children diagnosed with DLD (study group) and 30 typically developing children (control group). A total of 23.3% of the children in the study group are girls (*n* = 7), 76.7% are boys (*n* = 23), and the average age is 50.96 ± 9.77. Of the children in the control group, 36.7% were girls (*n* = 11), 63.3% were boys (*n* = 19), and the average age was 51.36 ± 9.89. There is no statistically significant difference in terms of gender and age averages between the two groups (*p* = 0.260; *p* = 0.875). It can be said that the groups are similar in terms of gender distribution and average age (Table 1).

**TABLE 1:** Comparison of the mean age and gender distribution of the groups.

Group	Age (month)			Gender			
	Mean ± SD	Test value	*p*	Girl	Boy	Test value	*p*
Study group (*n* = 30)	50.96 ± 9.77	−0.157^a^	0.875	7	23	1.270^b^	0.260
				23.3%	76.7%		
Control group (*n* = 30)	51.36 ± 9.89			11	19		
				36.7%	63.3%		

*Note: p *< 0.05.

^a^ Independent *t*‐test.

^b^ Chi‐square test results.

The study group demonstrated considerably lower scores in both the receptive and expressive language compared to the control group (*t*(58) = −7.899, *p *< 0.001, *d* = 2.0; *t*(58) = −8.316, *p *< 0.001, *d* = 2.1; Table 2).

**TABLE 2:** Comparison of ELD‐3: receptive and expressive language and nine‐factor scores results of children in the study and control groups.

Subtests/factors	Group	Mean ± SD	Test value (*t*)	*p*	*d*
ELD‐3: receptive language	Study (*n* = 30)	14.23 ± 5.53	−7.899^a^	**< 0.001**	**2.0**
	Control (*n* = 30)	23.80 ± 3.66			
ELD‐3: expressive language	Study (*n* = 30)	13.13 ± 6.39	−8.316^a^	**< 0.001**	**2.1**
	Control (*n* = 30)	25.00 ± 4.49			
Sensory seeking	Study (*n* = 30)	57.60 ± 12.16	−4.342^a^	**< 0.001**	**1.1**
	Control (*n* = 30)	68.80 ± 7.17			
Low endurance/tone	Study (*n* = 30)	39.76 ± 3.97	−0.896^a^	0.374	0.2
	Control (*n* = 30)	40.60 ± 3.17			
Oral sensory sensitivity	Study (*n* = 30)	35.86 ± 7.09	−1.851^a^	0.069	0.47
	Control (*n* = 30)	38.36 ± 2.07			
Inattention/distractibility	Study (*n* = 30)	25.70 ± 4.83	−5.140^a^	**< 0.001**	**1.3**
	Control (*n* = 30)	30.40 ± 1.30			
Poor registration	Study (*n* = 30)	35.40 ± 3.25	−1.404^a^	0.166	0.36
	Control (*n* = 30)	36.36 ± 1.90			
Sedentary	Study (*n* = 30)	16.80 ± 2.75	0.241^a^	0.810	0.06
	Control (*n* = 30)	16.63 ± 2.59			
Fine motor/perceptual	Study (*n* = 30)	7.66 ± 0.99	−13.743^a^	**< 0.001**	**3.5**
	Control (*n* = 30)	12.46 ± 1.63			

Sub‐factors	Group	Mean rank	Test value (*Z*)	*p*	
Sensory sensitivity	Study (*n* = 30)	29.00	−0.674^b^	0.500	0.1
	Control (*n* = 30)	32.00			
Emotionally reactive	Study (*n* = 30)	22.42	−3.592^b^	**< 0.001**	**0.5**
	Control (*n* = 30)	38.58			

*Note: p *< 0.05; *d*, effect size.

^a^ Independent *t*‐test.

^b^ Mann–Whitney *U* test.

When looking at the nine‐factor scores of the groups in Table 2, it is seen that the sensory seeking, inattention/distractibility, fine motor/perceptual, and emotionally reactive scores of the children in the study group differ significantly compared to the children in the control group (*t*(58) = −4.342, *p *< 0.001, *d* = 1.1; *t*(58) = −5.140, *p *< 0.001, *d* = 1.3; *t*(58) = −13.743, *p *< 0.001, *d* = 3.5; *z* = −3.592, *p *< 0.001, *d* = 0.5).

**FIGURE 1:**
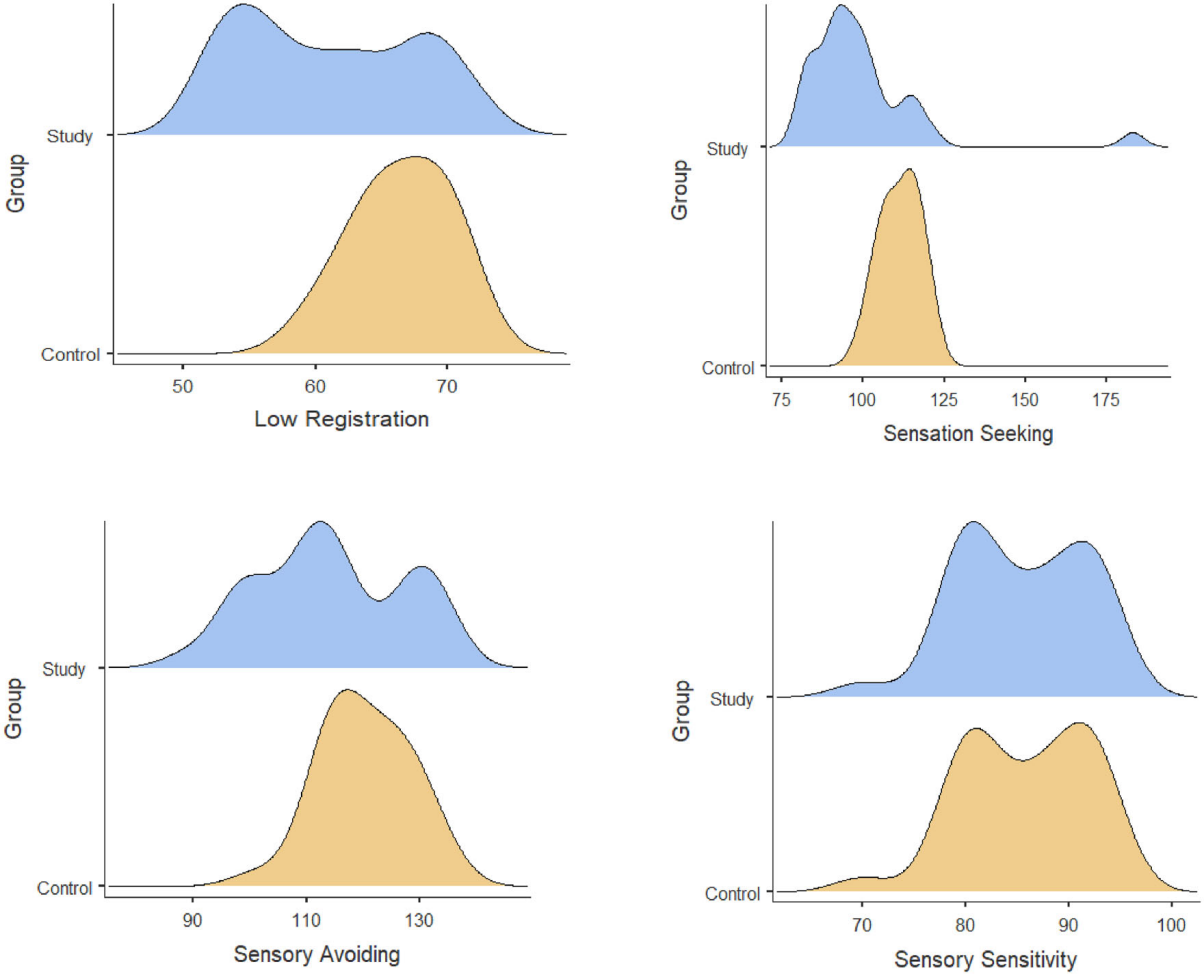
A graphic representation of the density of the groups’ four‐quadrant scores means.

When the quadrant scores of the groups are examined in Table 3, it is seen that the low registration, sensory avoiding, and sensation seeking scores of the children in the study group differ significantly compared to the children in the control group (*t*(58) = −4.025, *p *< 0.001, *d* = 1.0; *t*(58) = −2.237, *p* = 0.029, *d* = 0.5; *z* = −4.487, *p *< 0.001, *d* = 0.6). However, the two groups’ sensory sensitivity scores do not differ (*t*(58) = −0.233, *p* = 0.817, *d* = 0.06). Figure 1 gives a graphical representation of the density of the means scores of the groups in the four quadrants.

**TABLE 3:** Comparison of the four‐quadrant scores of children in the study and control groups.

Quadrant	Group	Mean ± SD	Test value (*t)*	*p*	*d*
Low registration	Study (*n* = 30)	60.96 ± 6.64	−4.025^a^	**< 0.001**	**1.0**
	Control (*n* = 30)	66.50 ± 3.54			
Sensory sensitivity	Study (*n* = 30)	85.36 ± 6.12	−0.233^a^	0.817	0.06
	Control (*n* = 30)	85.73 ± 6.06			
Sensory avoiding	Study (*n* = 30)	114.26 ± 12.77	−2.237^a^	**0.029**	**0.5**
	Control (*n* = 30)	120.36 ± 7.73			

Quadrant	Group	Mean rank	Test value (*Z*)	*p*	*d*
Sensation seeking	Study (*n* = 30)	20.40	−4.487^b^	**< 0.001**	**0.6**
	Control (*n* = 30)	40.60			

*Note: p *< 0.05; *d*, effect size.

^a^ Independent *t*‐test.

^b^ Mann–Whitney *U* test.

When Table 4 was examined, a significant relationship was found between ELD‐3: receptive language test scores and low registration (*r* = 0.425, *p* = 0.001), sensation seeking (*r* = 0.364, *p* = 0.004), inattention/distractibility (*r* = 0.356, *p* = 0.005), and fine motor/perceptual (*r* = 0.611, *p *< 0.001) scores. The study also found a correlation between scores on the ELD‐3: expressive language test scores and low registration (*r* = 0.339, *p* = 0.008), sensation seeking (*r* = 0.461, *p *< 0.001), sensory avoiding (*r* = 0.275, *p* = 0.033), sensory seeking (*r* = 0.300, *p* = 0.020), inattention/distractibility (*r* = 0.371, *p* = 0.004), and fine motor/perceptual (*r* = 0.568, *p *< 0.001).

**TABLE 4:** Correlation results between children's ELD‐3: receptive and expressive language scores and the nine‐factor and the four‐quadrant scores.

ELD‐3		Low registration	Sensation seeking	Sensory sensitivity	Sensory avoiding	Sensory seeking	Emotionally reactive	Low endurance/tone	Oral sensory sensitivity	Inattention/distractibility	Poor registration	Sensory sensitivity	Sedentary	Fine motor/perceptual
Receptive language	*r*	0.425^**^	0.364^**^	0.067	0.221	0.229	0.048	0.002	0.080	0.356^**^	0.116	−0.180	0.030	0.611^**^
	*p*	**0.001**	**0.004**	0.610	0.090	0.078	0.715	0.989	0.546	**0.005**	0.378	0.168	0.821	**< 0.001**
Expressive language	*r*	0.339^**^	0.461^**^	0.002	0.275^*^	0.300^*^	−0.012	−0.021	0.071	0.371^**^	0.031	−0.160	−0.098	0.568^**^
	*p*	**0.008**	**< 0.001**	0.986	**0.033**	**0.020**	0.926	0.873	0.592	**0.004**	0.811	0.222	0.455	**< 0.001**

*Note: r*, Pearson correlation.

* *p* < 0.05.

** *p* < 0.01.

## 4 Discussion

Sensory processing is integral to children's experience of activities that support learning. Whenever children display behavioral reactions to sensory stimuli that affect their engagement in everyday tasks, caregivers frequently adjust their activities or surroundings to respond to their sensory aversions or preferences (Little et al. 2017). A child with a sensory processing disorder finds it difficult to participate in daily routines as well as social and community activities. If a child has a sensory processing disorder, the child's participation functions in daily routines and social and community activities are impaired. Although these children are participating in school, they may be distracted by the intensity and multiple types of sensory stimuli in environments such as the classroom, cafeteria, and playground, and they may have trouble paying attention and mingling appropriately with other children (Kramer and Hinojosa 2010). In early childhood, differences in the child's sensory processing can impact the child's development of age‐appropriate learning, attention, motor skills, academic achievements, and social interactions (Baş and Yücel 2023). Studies have shown that children with ASD, ADHD, hearing impairment, and language impairment have differences in sensory processing skills compared to those without neurodevelopmental problems (Baş and Yücel 2023; Bharadwaj, Daniel, and Matzke 2009; Dunn 2007; Rani et al. 2023; Thye et al. 2018). However, Little et al. (2017) emphasize in their study on the general population that high scores in sensory processing patterns in children are not specific to ASD but are a reflection of children's ability to respond to environmental demands. Another study identified sensory processing problems that impacted daily life activities in children aged 2–7 who did not have ASD, intellectual disability, or auditory or visual impairments. Sensory over‐responsiveness was present in 60% of the children, sensory under‐responsiveness in 51%, sensory craving in 42%, sensory‐based motor disorder in 40%, and sensory discrimination disorder in 29% (Mulligan, Douglas, and Armstrong 2021). Our study showed differences in sensory processing skills between DLD and typically developing children who showed similar characteristics in terms of age and gender in the preschool period.

Children with DLD are at risk for developing speech and language disorders due to their difficulties regulating the amount of sensory input they receive and their impairments in the auditory processing process (Van der Linde, Franzsen, and Barnard‐Ashton 2013). It is stated that some children with language and speech disorders (e.g., reading difficulty, developmental language disorder) have difficulties with sensory processing tasks, especially auditory and visual processing (Hulslander et al. 2004; Van der Linde, Franzsen, and Barnard‐Ashton 2013). Simpson et al. (2020) reported that the sensory processing scores of 28 children with DLD and receiving special education were significantly lower than the normal range of the test. They also stated that these children's tactile sensitivity, sensory seeking/less responsiveness, auditory filtering, and weak/low energy were below the standard range, but taste/smell, movement, and visual/auditory sensitivity were within the normal range. Children with DLD have challenges with inattention/distractibility in 81.8% of cases, fine motor/perceptual difficulties in 72.7%, emotionally reactive in 63.6%, and sensory seeking in 54.5%, according to a similar study. Furthermore, there have also been reports indicating that children diagnosed with DLD exhibit differences in sensory processing skills compared to children diagnosed with ASD and ADHD. In comparison to children with DLD and ADHD, children with ASD demonstrated the lowest performance (Van der Linde, Franzsen, and Barnard‐Ashton 2013). Children with DLD in our study had very low sensory seeking, inattention/distractibility, fine motor/perceptual, and emotionally reactive scores. This condition occurs when children who are less sensitive to sensory responses ignore or do not respond to sensory stimuli in their environment. These children react to sensory stimuli in a weaker, indifferent, and unperceived way than they should. As a result, these children become slowpoke, lethargic, timid, and inattentive. However, the reduced response to sensory stimuli is not due to a lack of motivation but rather to a failure to recognize the possibilities of events (Miller et al. 2007). Children who are sensory seeking require more sensory stimulation than they should. Their constant movement and engaging in activities that provide more stimuli, like hopping, jumping, and careless behavior, can lead to confusion with ADHD (Miller et al. 2007; Van der Linde, Franzsen, and Barnard‐Ashton 2013).

It is stated that children suspected of childhood apraxia have significant differences in the area of behavioral and emotional responses compared to their peers. The scores of these children differed considerably, particularly in four quadrants: sensory sensitivity, sensory avoiding, low registration, and sensation seeking (Newmeyer et al. 2009). They state that sensitivity and avoidance may be seen together in children with ASD and ADHD because they exhibit behavioral characteristics such as high noticing/response (Dean et al. 2022). Delgado‐Lobete et al. (2020) found that children with developmental coordination disorder and ADHD had higher sensory quadrant scores compared to typically developing children. Sensory sensitivity and low registration were more common in children with developmental coordination disorder. ADHD children reported higher rates of low registration, sensory sensitivity, and sensation seeking. Another study found that children with poor motor coordination had an elevated chance of experiencing psychosocial and emotional issues, such as psychological issues, low self‐esteem, anxiety, and difficulty in social involvement (Blank et al. 2019). Casey (2016) states that most children with specific language disorders meet the criteria for some type of sensory processing difficulty in two or more quadrants. In our study, the three‐quadrant scores (low registration, sensory avoiding, and sensation seeking) of children with DLD were lower than those of typically developing children. This finding shows that these children's response to sensory stimuli is weak, or they need more intense stimuli. Therefore, the planning of assessment and intervention strategies for children with DLD should also consider their sensory processing skills.

Studies examining the relationship between language skills and sensory processing skills vary (Krüger et al. 2001; Newmeyer et al. 2009; Simpson et al. 2020). Simpson et al. (2020) found that there was no significant relationship between the language skills and sensory processing skills of children with DLD. Another study stated that there was a significant relationship between central auditory processing, language processing, and academic and sensory processing skills (Krüger et al. 2001). Newmeyer et al. (2009) revealed a substantial positive association between the severity of oral‐motor apraxia and the sensitivity score from the quadrants. Our research revealed a significant correlation between language skills and two or three quadrants (poor registration, sensation seeking, and sensory avoiding) as well as three factors (sensory seeking, inattention/distractibility, and fine motor/perceptual). Impaired modulation of sensory input can negatively affect the language processing process. When language disorders and sensory processing disorders occur together in a child, intervention in both areas is necessary (Taal et al. 2013).

## 5 Conclusion

It has been observed that the sensory processing skills of children with DLD differ in factors such as sensory seeking, inattention/distractibility, fine motor/perceptual, emotionally reactive factors, and quadrant scores such as low registration, sensation sensitivity, and sensory avoiding. In other words, children with DLD appear to have difficulties in some areas of sensory processing skills. Hence, with language intervention strategies for children experiencing language developmental disorders, it is essential to have early and precise diagnostic examinations to avert any challenges that may arise in subsequent stages of life. Thus, by using a multidisciplinary team approach, one may use the interplay between language and speech development and sensory processing skills to create a comprehensive intervention strategy. Based on the findings of our research, it is believed that this information will assist clinicians in identifying the most efficient intervention techniques for children with DLD.

The limitation of our study is that we could not examine the relationship between DLD‐related risk factors, different demographic variables, and sensory processing skills.

## Author Contributions

**Elife Barmak**: Software; investigation, conceptualization; methodology; writing–review and editing; resources. **Banu Baş**: Conceptualization; investigation; methodology; writing–review and editing; software; resources.

## Disclosure

Our article has not been presented anywhere before.

## Ethics Statement

Ethical permission was obtained from the Health Sciences Ethics Committee of Ankara Yıldırım Beyazıt University before the data were collected in the study (date: December 9, 2021; protocol no. 33).

## Conflicts of Interest

The authors declare no conflicts of interest.

### Peer Review

The peer review history for this article is available at https://publons.com/publon/10.1002/brb3.70105

## Data Availability

The data that support the findings of the study are available from the corresponding author, upon reasonable request.
